# Distinct Mitotic Functions of Nucleolar and Spindle-Associated Protein 1 (NuSAP1) Are Controlled by Two Consensus SUMOylation Sites

**DOI:** 10.3390/cells12212545

**Published:** 2023-10-30

**Authors:** Michela Damizia, Ludovica Altieri, Vincenzo Costanzo, Patrizia Lavia

**Affiliations:** 1 Institute of Molecular Biology and Pathology (IBPM), CNR National Research Council of Italy, 00185 Rome, Italy; michela.damizia@unitn.it (M.D.); ludovica.altieri@uniroma1.it (L.A.); vincenzo.costanzo@ibpm.cnr.it (V.C.); 2 Department of Biology and Biotechnology “Charles Darwin”, Sapienza University of Rome, 00185 Rome, Italy; 3 Department of Cellular, Computational and Integrated Biology (CIBIO), University of Trento, 38123 Trento, Italy

**Keywords:** NuSAP1, RANBP2, mitosis, microtubules, kinetochores, SUMOylation, chromosome segregation

## Abstract

Nucleolar and Spindle-Associated Protein 1 (NuSAP1) is an important mitotic regulator, implicated in control of mitotic microtubule stability and chromosome segregation. NuSAP1 regulates these processes by interacting with several protein partners. Its abundance, activity and interactions are therefore tightly regulated during mitosis. Protein conjugation with SUMO (Small Ubiquitin-like MOdifier peptide) is a reversible post-translational modification that modulates rapid changes in the structure, interaction(s) and localization of proteins. NuSAP1 was previously found to interact with RANBP2, a nucleoporin with SUMO ligase and SUMO-stabilizing activity, but how this interaction affects NuSAP1 activity has remained elusive. Here, we show that NuSAP1 interacts with RANBP2 and forms proximity ligation products with SUMO2/3 peptides in a RANBP2-dependent manner at key mitotic sites. A bioinformatic search identified two putative SUMO consensus sites in NuSAP1, within the DNA-binding and the microtubule-binding domains, respectively. Site-specific mutagenesis, and mitotic phenotyping in cell lines expressing each NuSAP1 mutant version, revealed selective roles of each individual site in control of NuSAP1 localization and in generation of specific mitotic defects and distinct fates in daughter cells. These results identify therefore two new regulatory sites for NuSAP1 functions and implicate RANBP2 in control of NuSAP1 activity.

## 1. Introduction

Human Nucleolar and Spindle-Associated Protein 1 (NuSAP1) is a microtubule-binding protein with regulatory roles during mitosis. It is expressed in a cell cycle-regulated manner, first appearing in G2-phase cells, during which it is predominantly nucleolar, and reaches peak levels in mitosis when it localizes at the mitotic spindle, particularly at microtubule growing ends (plus ends) and partly at chromosomes [1].

While the function of NuSAP1 in nucleoli is not clarified, converging lines of evidence indicate important roles in mitosis via regulation of the spindle microtubules. Indeed, NuSAP1 stabilizes microtubules during spindle assembly via two main mechanisms: microtubule protection against destabilization and microtubule cross-linking in asters and networks [2,3]. These activities can be formally separated for being differentially sensitive to nuclear import receptors that bind NuSAP1 in its nuclear localization signal, which flanks the microtubule-binding domain: the binding of importin beta alone selectively prevents NuSAP1 function in aster formation, while the binding of importin alpha/importin 7 dimer blocks NuSAP1 activity in microtubule elongation, though not affecting aster formation [2,3]. Thus, nuclear import receptors bind NuSAP1 in ways that inhibit distinct functions. The inhibition is released in the proximity of the GTPase RAN, which binds to importin vectors and determines the release of free, biologically active NuSAP1.

NuSAP1 also interacts with microtubule motor proteins. It binds to and counteracts the activity of Mitotic Centromere-Associated Kinesin (MCAK), a kinesin with microtubule-depolymerizing activity. By attenuating MCAK activity at kinetochores, NuSAP1 stabilizes their attachment to microtubules [4]. NuSAP1 also modulates the activity of the Kinesin-like DNA-binding protein Kid, a plus end-directed chromokinesin, in control of chromosome orientation and oscillations during alignment prior to the onset of chromosome segregation [5]. Thus, NuSAP1 regulates the mitotic apparatus both via direct regulation of microtubule stability and by modulating centromere/kinetochore kinesins.

NuSAP1 harbors an SAP domain (named after the Scaffold attachment factors A/B, the chromatin condensation factor Acinus and the protein inhibitor of activated STAT, PIAS, first found to contain it) in its N-terminal region [6]. The SAP domain is common among proteins acting in transcription, DNA repair and RNA processing. It is proposed to regulate NuSAP1 interactions with chromatin and microtubule stabilization around chromosomes [6].

To exert these functions, both NuSAP1 abundance and spatial distribution need be stringently regulated. Indeed, NuSAP1 overexpression causes microtubule bundling and prevents their depolymerization in vitro [1], resulting in spindle misfunction, that can originate genomic imbalance in daughter cells. NuSAP1 depletion also yields abnormal spindles with unstable microtubules that drive chromosome misalignment and faulty cytokinesis [2]. NuSAP1 expression is often deregulated in cancers of different origin [7], in which profiling studies have pinpointed it among differentially expressed genes, often as part of key signatures. Interestingly, an NuSAP1 mutation generating a truncated protein lacking the MCAK-binding domain has been recently identified in subjects affected by microcephaly [8], a neurodevelopmental disorder with reduced brain size, often reflecting defects in mitotic divisions of neuronal progenitors [9].

NuSAP1 protein levels are controlled via a KEN box targeted by the APC/C ubiquitin ligase, which conveys it to massive degradation in late telophase and cytokinesis [10]. NuSAP1 also moderately turns over during spindle assembly, as its release from importin vectors render it accessible to the ubiquitination/degradation machinery [11]. Thus, importin beta loads NuSAP1 onto microtubules and protects it from the ubiquitin/proteasome system [12]; concomitantly, however, it inhibits NuSAP1′s activity in the interaction [2,3].

One study has shown that NuSAP1 co-immunoprecipitates with RANBP2 [13], the largest nucleoporin in nuclear pores. RANBP2 is endowed with E3 ligase activity for protein conjugation with SUMO (Small Ubiquitin-like MOdifier) [14]. SUMOylation is a reversible post-translational modification that modulates rapid switches in protein structure, interaction(s) and localization, relevant to processes entailing dynamic responses, e.g., DNA replication, DNA damage repair and mitosis [15,16,17]. Among the isoforms of human SUMO peptides, SUMO2 and SUMO3 are 96% identical and together represent the most abundant species, henceforth, collectively referred to as SUMO2/3. Growing evidence highlights the importance of SUMOylation during mitosis. Several mitotic factors are SUMOylation substrates, many at kinetochores [18,19,20]. Kinetochores also recruit SUMO-conjugating and -deconjugating enzymes [19,21,22,23], suggesting that SUMO modification cycles take place therein. Some factors are also SUMOylated at the mitotic spindle [24].

RANBP2 can directly conjugate proteins with SUMO via its SUMO E3 ligase domain, as in the case of borealin [25]. In addition, via a SUMO-interacting motif (SIM) overlapping the E3 domain, RANBP2 can interact with, and stabilize, proteins SUMOylated by other ligases, the best characterized of which is RANGAP1, a RAN GTPase regulator SUMOylated by the SUMO ligase UBC9 [26,27]. The association of RANBP2, RANGAP1-SUMO and UBC9 gives rise to a multimeric complex (the RRSU complex) that has increased SUMO-conjugating activity compared to RANBP2 alone. In interphase, RRSU aids transport across nuclear pores [28,29]. After nuclear envelope breakdown, RANBP2 and RANGAP1-SUMO co-localize at the spindle microtubules and at microtubule-attached kinetochores [30,31] and can regulate the SUMO-conjugated state of proteins therein. Several studies have shown that RANBP2 silencing is detrimental to proper mitotic progression and chromosome segregation [30,32,33,34], which reflects, at least in part, its activity in regulation of SUMOylated mitotic factors at their site of action [25,35,36,37,38].

The identification of RANBP2 in the NuSAP1 co-IP, and subsequent studies, have led to suggest that NuSAP1 acts as a scaffold bridging the RRSU complex to proteins with which NuSAP1 interacts, e.g., TCF4 [39] and ATR [40]. It remains unclear, however, whether NuSAP1 can itself be SUMOylated and, if so, whether that affects its functions. Here, we find that RANBP2 regulates NuSAP1 abundance. We also find that NuSAP1 forms proximity ligation products with SUMO2/3 peptides in a RANBP2-dependent manner, suggesting that NuSAP1 can be a target of SUMO2/3 conjugation. Bioinformatic tools identified putative SUMO-conjugatable lysine residues in NuSAP1, respectively, in the SAP and the microtubule-binding domains. Mutation of each lysine selectively destabilized NuSAP1′s association with chromatin and with microtubules, respectively, yielding specific mitotic defects and distinct cell fates after mitosis. These data suggest that the newly identified SUMOylatable lysines represent previously unidentified regulatory sites for NuSAP1′s functions in mitotic processes.

## 2. Materials and Methods

### 2.1. Cell Cultures and Synchronization

Human HeLa cells (American Tissue Culture Collection, Manassas, Virginia, http://www.atcc.org, CCL-2) were grown in DMEM supplemented with 10% fetal bovine serum, 2% L-glutamine, 2.5% HEPES and 2% penicillin/streptomycin at 37 °C in 5% CO_2_. Cells were synchronized in mitosis by adding (i) either S-trityl-L-cysteine (STLC, 2 μM, 18 h; Sigma Aldrich, St Louis, MO, USA) to inhibit kinesin Kif11 and induce prometaphase arrest or (ii) RO3306 (9 μM, Sigma Aldrich, 20 h) to inhibit CDK1 and arrest cells at the G2/M transition. After either protocol, cultures were released in fresh medium to progress through mitosis, and detaching prometaphase and metaphase cells were collected via mechanical shake-off.

### 2.2. RNA Interference (RNAi)

The following small interfering (si)RNA duplexes were used: RANBP2: 5′-GGACAGUGGGAUUGUAGUGtt-3′ (Ambion, Carlsbad, CA, USA); NuSAP1: 5′-GCACCAAGAAGCUGAGAAUtt-3 (Sigma Aldrich); and GL2 control (against the firefly luciferase, no target in mammalian cells): 5′-CGUACGCGGAAUACUUCGAtt-3′. Final siRNA concentrations were 150 nM for RANBP2 and 50 nM for GL2 and NuSAP1. siRNAs were diluted in serum-free OptiMem and transfected using Lipofectamine RNAiMAX (both from Invitrogen, Waltham, MA, USA).

### 2.3. Generation of Stable Cell Lines Expressing NuSAP1 Mutants

Expression vectors for NuSAP1-EGFP and mutant derivatives were engineered in the enhanced transposon-based piggyBac vector. The vector, epB-Bsd-TT EGFP, derived from epB-Puro-TT [41], carries a blasticidin resistance gene and a tetracycline-responsive promoter element (*TRE Tight*) followed by a multi-cloning site linked to an EGFP tag. To generate epB-Bsd-TT-NuSAP1-EGFP, the NuSAP1 sequence (NCBI ref BC001308) was modified as follows: (i) introduction of neutral mutations (420C < T; 422A < G; 424A < G; 426T < C; 428G < A430) in all constructs to render the exogenous transcripts resistant to NuSAP1-specific siRNAs, and (ii) introduction of specific mutations (131A < G and 944A < G) in individual constructs to obtain NuSAP1^K44R^ and NuSAP1^K316R^ mutants, respectively. siRNA-resistant NuSAP1 sequences were cloned via gene synthesis in frame with EGFP. HeLa cells were co-transfected with each construct and with the hypb7 plasmid, encoding the transposase gene, using Lipofectamine (Invitrogen). A total of 24 h after transfection, the medium was replaced with Tet-free DMEM supplemented with 3 μg/mL blasticidin-S hydrochloride (Sigma Aldrich). Blasticidin-S-resistant foci were isolated, expanded and tested for expression after administration doxycycline hyclate (dox, 1 μg/mL, 9 h, Santa Cruz Biotechnology, Heidelberg, Germany).

### 2.4. Immunofluorescence (IF)

Cells grown on coverslips coated with poly-L-lysine (Sigma Aldrich) were fixed as follows: (a) 3.7% paraformaldehyde (PFA, Sigma Aldrich)/30 mM sucrose/PBS (10 min), permeabilization in 0.1% Triton X-100 (5 min) and incubation with glycine 0.1 M (10 min); (b) incubation in PHEM (60 mM PIPES, 25 mM HEPES, 10 mM EGTA, 2 mM MgCl_2_, pH 6.9) containing 0.3% Triton X-100 (6 min), followed by PFA fixation as above. Blocking and incubation with antibodies (in PBS containing 0.05% Tween-20/3% bovine serum albumin) were at room T°. Primary antibodies are in Table S1. Secondary antibodies were conjugated to fluorescein isothiocyanate (FITC), Cy3, Alexa-Fluor 647 (Cy5) or 7-amino-4-methylcoumarin-3- acetic acid (AMCA) (all from Jackson Immunoresearch Laboratories, West Grove, PA, USA). DNA was stained with 0.1 µg/mL 4,6-diamidino-2-phenylindole (DAPI, Sigma Aldrich). Coverslips were mounted in Vectashield (Vector Laboratories, Newark, CA, USA).

### 2.5. Proximity Ligation Assays (PLAs)

We developed both intermolecular and intramolecular PLA assays. In intermolecular PLAs, primary antibodies were directed against two proteins of interest, and secondary antibodies were linked to complementary single-stranded oligonucleotides. If the proteins interacted or were located in close proximity, the complementary oligonucleotides annealed and ligated upon addition of an exogenous ligase. The ligation product was then amplified according to the rolling cycle model and finally recognized by a fluorescent probe, which represented the PLA signal. In intramolecular PLAs, primary antibodies were directed against a protein of interest and a peptide that may be conjugated to it; in this case, SUMO2/3 peptides. Secondary antibodies were added and the reaction continued as for intermolecular PLA. In technical terms, cells were fixed, blocked and incubated (1 h, room T°) with primary antibodies (Table S1). Secondary antibodies conjugated to PLA probes (PLA Duolink In Situ PLA Probe, Anti-Rabbit PLUS, DUO92002, and Duolink In Situ PLA Probe Anti-Mouse MINUS, DUO92004, Sigma Aldrich) were added (diluted 1:5 in PBS containing 0.05% Tween-20 and 3% bovine serum albumin) and incubated in a pre-heated humidity chamber (1 h, 37 °C). Ligation (30 min, 37 °C) and amplification were performed at 37° following the Olink Bioscience protocol using two alternative Duolink Detection kits (Sigma-Aldrich): DUO92007 orange, containing a fluorescent detection probe (λ excitation 554 nm, λ emission 576 nm) visualized under the Cy3 filter, or DUO92008 red (λ excitation 594 nm and λ emission 624 nm), visualized under the Texas Red filter. PLA signals were localized via staining either for DNA using DAPI or for mitotic markers (Table S1) and quantified using the “spot count” function of NIS-Elements AR 5.02 software (Nikon, Amstelveen, Netherlands) in whole cells or in regions of interest (RoI), selected using DAPI for the DNA, CREST staining for kinetochores and tubulin staining for the spindle.

### 2.6. Time-Lapse Videorecording

Cells were seeded in 8-well μ-slide (80821, IbiTreat; Ibidi, Gräfelfing, Germany). During recording, cultures were kept at 37 °C in a T°- and CO_2_-controlled stage incubator (Okolab, Napoli, Italy) and recorded (48 h) under a Ti Eclipse automated inverted microscope (Nikon) equipped with a DS-Qi1MC camera, an Intensilight C-HGFIE lamp and NIS-Elements 3.1 software (Nikon). Images were taken using either a phase contrast (60 × 0.7 NA) or an immersion oil (60 × 1.4 NA) objective: phase every 15 min, GFP fluorescence every 60 min.

### 2.7. Mitotic Spindle Depolymerization and Microtubule Regrowth Assays

In spindle depolymerization assays, NuSAP1^WT^, NuSAP1^K44R^ and NuSAP1^K316^ cell lines were first subjected to endogenous NuSAP1 silencing, then induced to express their respective NuSAP1 versions (DOX, 9 h), incubated on ice under limiting conditions (15 min) and fixed in PFA for tubulin IF staining. In microtubule regrowth assays, cells were placed on ice for 40 min to achieve complete depolymerization, then incubated at 37 °C (1 min). After washing 2× in PTEMF buffer (20 mM PIPES, 10 mM EGTA, 1 mM MgCl_2_ in dH_2_O) to preserve resistant microtubules, cells were fixed (3.7% PFA, 0.2% Triton X-100 in PTEMF) and processed for IF.

### 2.8. High-Resolution Microscopy and Image Acquisition

Fixed samples were analyzed under a Nikon Eclipse 90i microscope equipped with a Qicam Fast 1394 CCD camera (Qimaging). Single cell images were taken under an immersion oil 100× objective (NA 1.3) and fields under a 40× objective (NA 0.75). Image analyses were performed using NIS-Elements AR 5.02 software modules (Nikon). A 3D deconvolution (0.4 μm z-serial optical sections) was performed using the AutoQuant deconvolution module. Image projections from z-stacks were created using the Maximum Intensity Projection (MIP) for quantitative analyses. IF signals were quantitatively analyzed on nd2 format files with automated external background correction, and the sum intensity of signals within selected RoIs was measured. PLA signals were automatically counted on 3D-acquired images, then processed by MIP activating the “spot detection” and “count objects” tools of NIS-Element AR 5.02 [42]. All figures represent MIP images unless specified otherwise. Images were processed with Adobe Photoshop CS 8.0.

### 2.9. Cell Extract Preparation and Western Immunoblotting (WB)

Cells were lysed in RIPA buffer (50 mM Tris-HCl pH 8, 150 mM NaCl, 1% NP40, 1 mM EGTA, 1 mM EDTA, 0.1% SDS, 0.25% sodium deoxycholate) supplemented with protease (Roche) and phosphatase (PhoSTOP, Roche, Basel, Switzerland) inhibitors. For subcellular fractionation, cell lines silenced for the endogenous NuSAP1 and induced to express NuSAP1^WT^, NuSAP1^K44R^ or NuSAP1^K316^ were synchronized in mitosis as above and collected via shake-off. After washing in PBS/protein inhibitor cocktail, cells were centrifuged, resuspended in hypotonic buffer (10 mM TrisHCl, pH7.4; 10 mM KCl; 1.5 mM MgCl_2_; 0.5 mM DTT), incubated on ice (10 min), gently homogenized in a Dounce homogenizer and centrifuged at 1500× *g*, 4 °C, 15 min. The supernatant represented the cytosolic fraction. The pellet, resuspended in TM buffer (50 mM TrisHCl, pH 7.4; 5 mM MgCl_2_) supplemented with DNAse (2000 U/mL), RNAse (200 μg/mL), and protein inhibitors, was put on a rotating wheel (overnight, room T°) and further incubated on the wheel the next day after re-adding fresh DNAse/RNase mix for another 2 h. After centrifugation (1000× *g*, 15 min), the supernatant representing the chromatin-bound fraction was collected. The pellet was resuspended under increasing ionic strength: first in 0.5 M buffer (0.5 M NaCl, 50 mM Tris HCl pH 7.4, 0.2 nM MgCl_2_, 1% TritonX-100), then in buffers containing 1 M and 2 M NaCl. The final insoluble pellet was resuspended in Laemmli buffer (Bio-Rad, Hercules, CA, USA) to obtain the chromosomal scaffold fraction. The fractions were probed using the following antibodies: tubulin and RANBP1 (mitotic cytosol); RCC1, a chromatin and histone-binding protein (chromatin and 0.5 M–1 M fractions); topoisomerase 2 alpha (TOP2A, scaffold fraction). Whole cell or fractionated extracts were separated through SDS-PAGE and transferred to nitrocellulose filters (Protran BA83, Whatman, Maidstone, UK) in a semi-dry or wet system (BIORAD). Blocking and antibody incubations were in TBS (10 mM Tris-HCl pH 7.4, 150 mM NaCl) containing 0.1% Tween 20 and 5% low-fat milk (1 h, room T°). Primary antibodies are listed in Table S1. HRP-conjugated antibodies (Santa Cruz Biotechnology) were revealed using the ECL system (GE Healthcare, Chicago, IL, USA) on Hyperfilm-ECL films (GE Healthcare, Chicago, IL, USA).

### 2.10. Statistical Analysis

Data were analyzed using GraphPad Prism 8. The following tests were employed: χ^2^ test or multiple χ^2^ test to compare the frequency of categorical variables (contingency tables), represented by histograms; Student’s *t* test to compare mean values between two independent groups; Mann–Whitney non-parametric test, or Dunn’s test, to compare continuous values, which in most experiments had a non-normal distribution across samples. The distribution of values measured in single cells is represented in dot plots.

## 3. Results

### 3.1. NuSAP Interacts with RANBP2 and RANGAP1 at Microtubule Growing Ends during Mitosis

**Figure 1:**
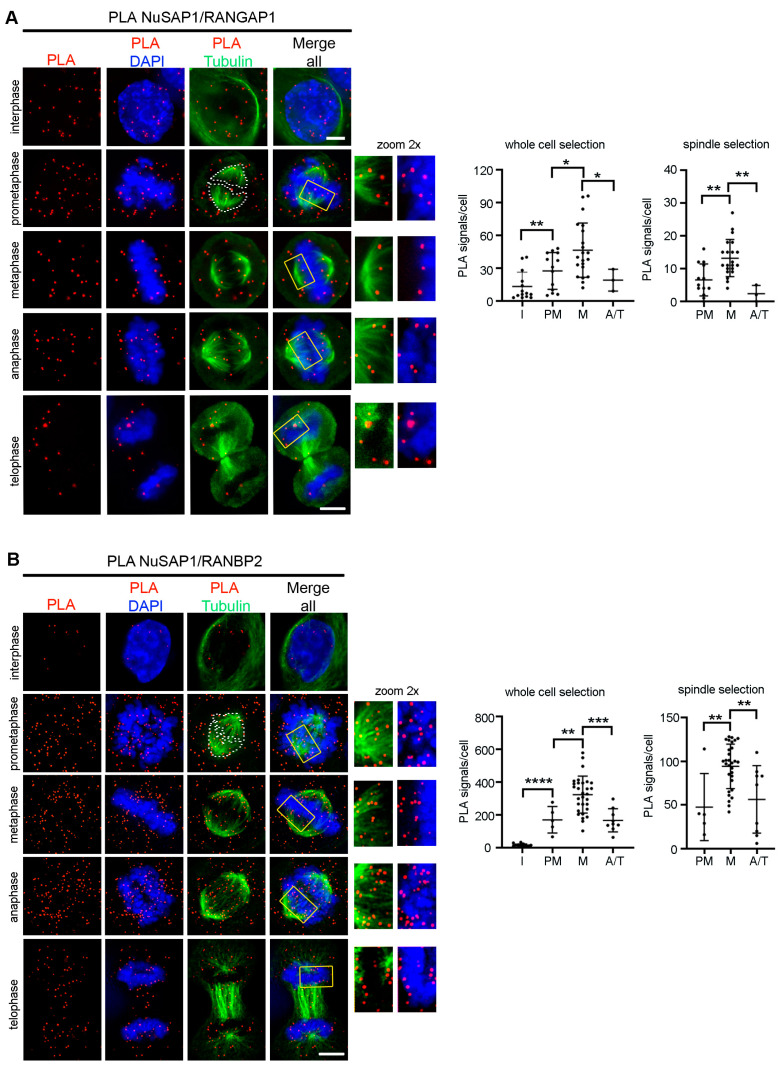
NuSAP1 forms PLA products with both RANGAP1 and RANBP2 during mitosis. (**A**) NuSAP1-RANGAP1 products were visualized to test the PLA protocol effectiveness. (**B**) NuSAP1-RANBP2 PLA products during mitotic progression. In both (**A**,**B**) reactions, a fraction of PLA signals localize at microtubule plus ends in contact with chromosomes (the framed insets, 2× zoom, show PLA signals merged with either microtubules or DAPI). Bars, 5 µm. The graphs (right) show PLA signals/cell-automated quantification in the indicated mitotic stages, selecting either whole cells, or tubulin-stained microtubules, as the RoI (dashed area in the exemplifying prometaphase). A significant (*, *p* < 0.05), highly significant (**, *p* < 0.01), or extremely highly significant (***, *p* < 0.001; ****, *p* < 0.0001) increase was detected in metaphase compared to other stages, both at the spindle level and in whole cells (Mann–Whitney test, at least 50 analyzed cells for both NuSAP1-RANGAP1 and NuSAP1-RANBP2 pairs, 2 experiments).

Both NuSAP1 and RANBP2 comprise fractions localizing at the mitotic spindle and kinetochores (see introduction). Given that NuSAP1 and RANBP2 were previously found to interact in co-immunoprecipitation assays [13], we sought to gain spatial and temporal information on their interaction. To that aim we used the PLA methodology and also re-assessed the NuSAP1-RANGAP1 pair as a previously characterized PLA interaction [13]. Before nuclear envelope breakdown, NuSAP1 PLA products with both RANGAP1 and RANBP2 were rare. PLA products remarkably increased in mitotic cells, for both the NuSAP1-RANGAP1 (Figure 1A) and the NuSAP1-RANBP2 pairs (Figure 1B); thus, both interactions are genuinely mitotic. The graphs quantify PLA signals/cell in mitotic stages compared to interphase: for both reactions, PLA signals increased during early mitosis and peaked in metaphase, taking either whole cells or microtubules as the region of interest (RoI). A large proportion of NuSAP1-RANGAP1 PLA signals were widespread throughout mitotic cells, while a small, yet well-defined, fraction localized at microtubules and at the interface between microtubules and chromosomes, consistent with the spatial distribution observed in a previous study [13]. We observed a similar proportion of localized vs. widespread PLA products for the NuSAP1-RANBP2 pair (Figure 1B). The PLA signals for both reactions decreased in telophase, with the latest visible signals associated with the central spindle and around decondensing chromosomes. Based on these data, we conclude that the PLAs depict genuine NuSAP1 ligation products with the RRSU complex components at key mitotic sites.

### 3.2. RANBP2 Stabilizes NuSAP1 at Microtubule plus Ends during Spindle Assembly

**Figure 2:**
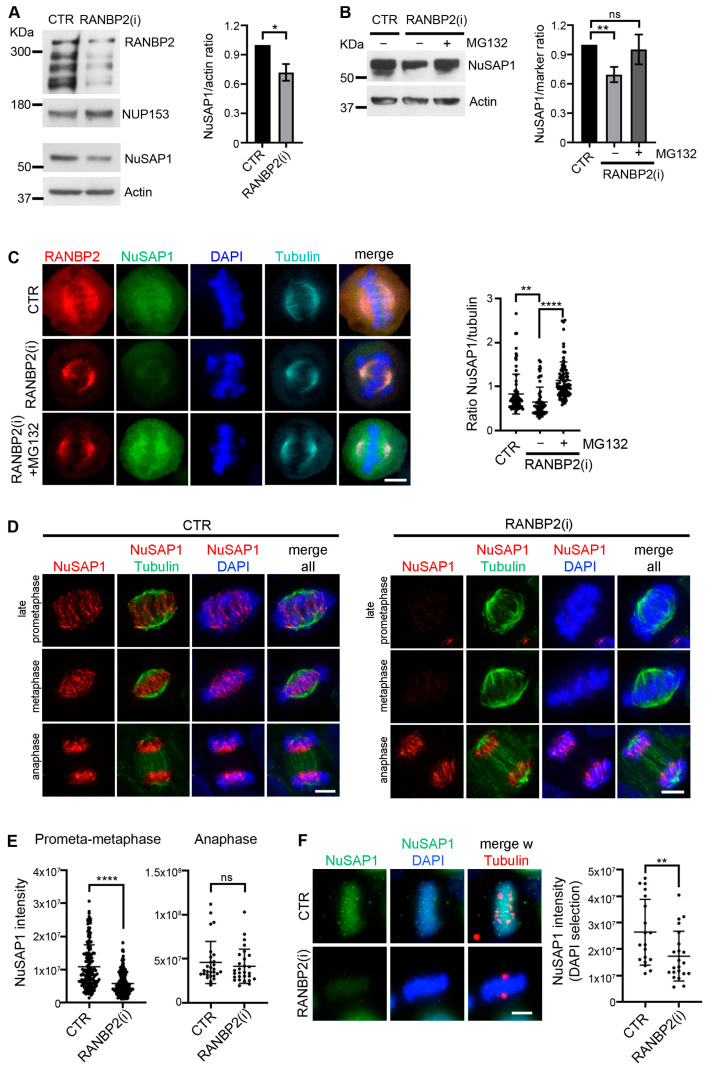
RANBP2 silencing down-regulates NuSAP1 abundance. (**A**) NuSAP1 abundance significantly decreases in RANBP2-silenced compared to control cultures (CTR) treated with neutral siRNA (*, *p* < 0.05, Student’s *t* test). Cultures treated with control or RANBP2-specific siRNAs were synchronized and collected via shake-off to obtain prometaphase and metaphase-enriched populations. Parallel gels were loaded with identical amounts of mitotic protein extract (25 μg/lane): RANBP2 silencing was verified on 6% SDS-PAGE using the nucleoporin NUP153 to control protein loading; NuSAP1 was analyzed on a twin 11% SDS-PAGE and controlled using actin. The graph shows the mean of densitometric values from 4 experiments. (**B**) MG132 rescues NuSAP1 abundance in RANBP2-silenced cells. The graph represents the mean of densitometric values from 3 experiments, with a highly significant (**, *p* < 0.01, panel (**B**)) decrease in NuSAP1 abundance in RANBP2-silenced vs. control cells, whereas MG132 addition to RANBP2-silenced cultures abolishes that difference (ns, non-significant). (**C**) NuSAP1 IF analysis in control and RANBP2-silenced cells, with or without MG132 addition. Cells were fixed with PFA to preserve the entire protein pool. The graphs quantify NuSAP1 IF signals normalized to tubulin. At least 65 cells/sample (2 experiments) were statistically analyzed (multi-comparison Dunn’s test **, *p* < 0.005; ****, *p* < 0.0001). (**D**) IF analysis of detergent-resistant NuSAP1 in RANBP2-silenced (right panel) and control (left panel) mitotic cells after PHEM/Triton solubilization prior to fixation: RANBP2 silencing substantially decreases NuSAP1 signals at microtubule ends until anaphase, after which NuSAP1 appears to be stabilized. (**E**) Quantification of NuSAP1 signal intensity in the indicated stages (whole cell selection). At least 200 cells per sample (5 experiments) were statistically analyzed (Mann–Whitney test: ****, *p* < 0.0001; ns, non-significant). (**F**) After microtubule depolymerization (30 min on ice) NuSAP1 localizes entirely to chromatin in control cells (top row); RANBP2 silencing prevents this association (bottom row). The graph quantifies NuSAP1 signal intensity/cell on DAPI selection. Mann–Whitney test, **, *p* < 0.01. Bars, 5 µm.

To assess the significance of NuSAP1-RANBP2 PLA interactions, we analyzed NuSAP1 in cultures lacking RANBP2 after siRNA-mediated knockdown compared to control cultures, interfered with neutral siRNA targeting the firefly luciferase gene, GL2 [37,38]. Cultures were synchronized using STLC, an inhibitor of kinesin KIF11, to arrest cells in prophase, then released to progress into mitosis and collected via mechanical shake-off to obtain cell populations enriched in late prometaphase and metaphase stages, when NuSAP1-RANBP2 PLA products peak (Figure 1). We found that NuSAP1 abundance decreased in RANBP2-silenced compared to control cells in Western assays (Figure 2A). The decrease was prevented by adding the proteasome inhibitor MG132 (Figure 2B).

NuSAP1 has a dynamic localization during mitosis [1]. We wondered whether RANBP2 silencing affected any particularly sensitive NuSAP1 fraction. Before addressing that question, we preliminarily examined NuSAP1 using immunofluorescence (IF) under conditions that reveal differentially soluble fractions [1]. Treatment with detergent prior to fixation depicted a resistant NuSAP1 fraction at microtubule plus ends (Figure S1A,C). When using direct paraformaldehyde (PFA) as the fixation agent, NuSAP1 was also visible along the spindle microtubules (Figure S1B,D), indicating that NuSAP1 is more soluble along the microtubule length than the growing end-associated fraction. When we examined RANBP2-silenced mitotic cells fixed with PFA, we found a highly significant decrease in the overall NuSAP1 signal intensity, which was counteracted by adding MG132, consistent with the Western data (Figure 2C).

In RANBP2-silenced cultures subjected to detergent solubilization prior to fixation, the detergent-resistant NuSAP1 fraction associated with microtubule plus ends practically disappeared (Figure 2D). Unexpectedly, however, that fraction remained unaffected after anaphase (quantified in Figure 2E).

Based on these data, RANBP2 contributes to stabilize NuSAP1 at microtubule plus ends while microtubules are engaged in chromosome search-and-capture and the spindle checkpoint is on, a process during which NuSAP1 is known to be phosphorylated by cyclin B/CDK1 [43]. When the SAC is satisfied, NuSAP1 becomes dephosphorylated and this is reported to stabilize its binding to tubulin [43]. We find that at this point it remains stably associated with the microtubule–chromosome interface regardless of RANBP2 presence or absence. We next examined mitotic cells devoid of microtubules after extensive depolymerization on ice: under these conditions, NuSAP1 was massively recruited to chromosomes in control cells, yet RANBP2 silencing yielded a complete disappearance of the NuSAP1 signal (Figure 2F). These experiments show that RANBP2 anchors NuSAP1, an unstable protein, at microtubule plus ends and concomitantly protects it from premature proteasome-dependent degradation during spindle assembly.

### 3.3. NuSAP1 Forms PLA Products with SUMO2/3 Peptides in a RANBP2-Dependent Manner

**Figure 3:**
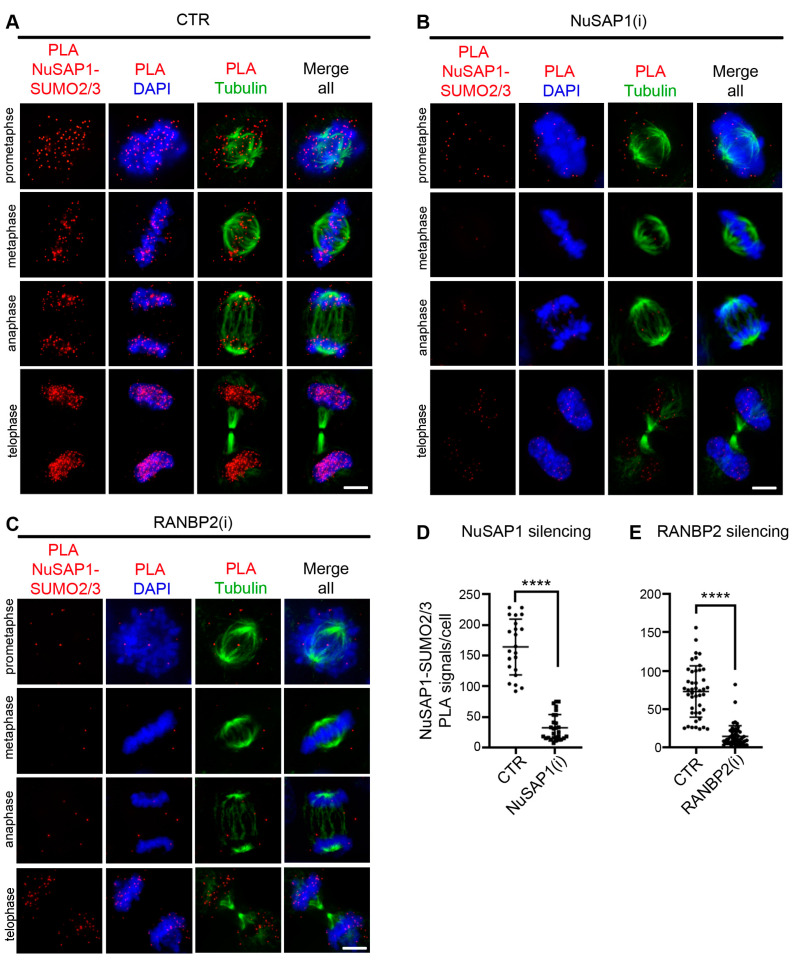
NuSAP1-SUMO2/3 PLA products form in an RANBP2-dependent manner. (**A**) In PHEM/Triton-solubilized controls (CTR, treated with neutral siRNAs), NuSAP1-SUMO2/3 PLA signals accumulate at the microtubule/kinetochore interface during progression towards metaphase, then progressively localize around chromosomes in anaphase and telophase. (**B**) NuSAP1-SUMO2/3 PLA signals fully disappear in cells treated with NuSAP1-specific RNAs. PLA signals from one representative experiment are quantified in the graph in (**D**): 30 analyzed cells per sample, Mann–Whitney test, ****, *p* < 0.0001. The same trend, with same statistical significance, was reproduced in an independent repeat with different basal intensity. (**C**) RANBP2 silencing abolishes NuSAP1-SUMO2/3 PLA signals in all mitotic stages. PLA signals are quantified in (**E**). At least 50 cells/sample were analyzed (3 experiments). Mann–Whitney test, **** *p* < 0.0001. Bars, 5 µm.

The finding that RANBP2 forms PLA products with NuSAP1 and stabilizes NuSAP1 at microtubule plus ends raises the question of whether NuSAP1 might itself be SUMOylated during mitosis. SUMOylation is a highly dynamic modification, which usually concerns a small fraction of target proteins, often with a high turnover and a restricted subcellular localization, which make its detection difficult under physiological conditions. The intramolecular PLA technique has been developed to circumvent these issues and has been successfully employed in several studies to visualize in situ ligation products formed by any given protein and SUMO peptides attached to it, avoiding any alteration of the original ratio of target protein to modifying SUMO peptides, but amplifying their detection [37,38,44,45]. Using this technique, we visualized PLA products formed by endogenous NuSAP1 and SUMO 2/3 peptides. In control cells, PLA signals progressively concentrated at microtubule plus ends and at the interface with chromosomes during progression towards chromosome alignment, particularly evidently in metaphase, when tension is highest (Figure 3A), and eventually to the decondensing chromosomes during anaphase. NuSAP1 silencing prevented the formation of PLA products with SUMO2/3 in all stages (Figure 3B, quantified in Figure 3D): this control indicates that the ligation is specific of NuSAP1 and is not due to SUMO peptide binding to neighboring proteins. After RANBP2 silencing, NuSAP1-SUMO2/3 PLA signals virtually disappeared throughout mitotic progression (Figure 3C,E), with only rare signals remaining visible. These data indicate that NuSAP1-SUMO2/3 products prevalently form at sites of microtubule interactions with chromosomes and that RANBP2 regulates their abundance.

### 3.4. Mutation of NuSAP1 Residues K44 and K316 Impair PLA Products with SUMO2/3 and Affect NuSAP1 Localization in Mitotic Cells

At this point, we searched for SUMO consensus motifs in the NuSAP1 using two bioinformatic tools: GSP-SUMO [46] and SUMO-plot [47]. Both searches identified several putative SUMOylatable lysine residues with varying probability scores according to the algorithm-based prediction employed by each program. Among the high-probability lysine residues consistently identified with both tools, two had a particularly interesting localization: K44 and K316, falling in the SAP- and the microtubule-binding domains, respectively (Figure S2A).

**Figure 4:**
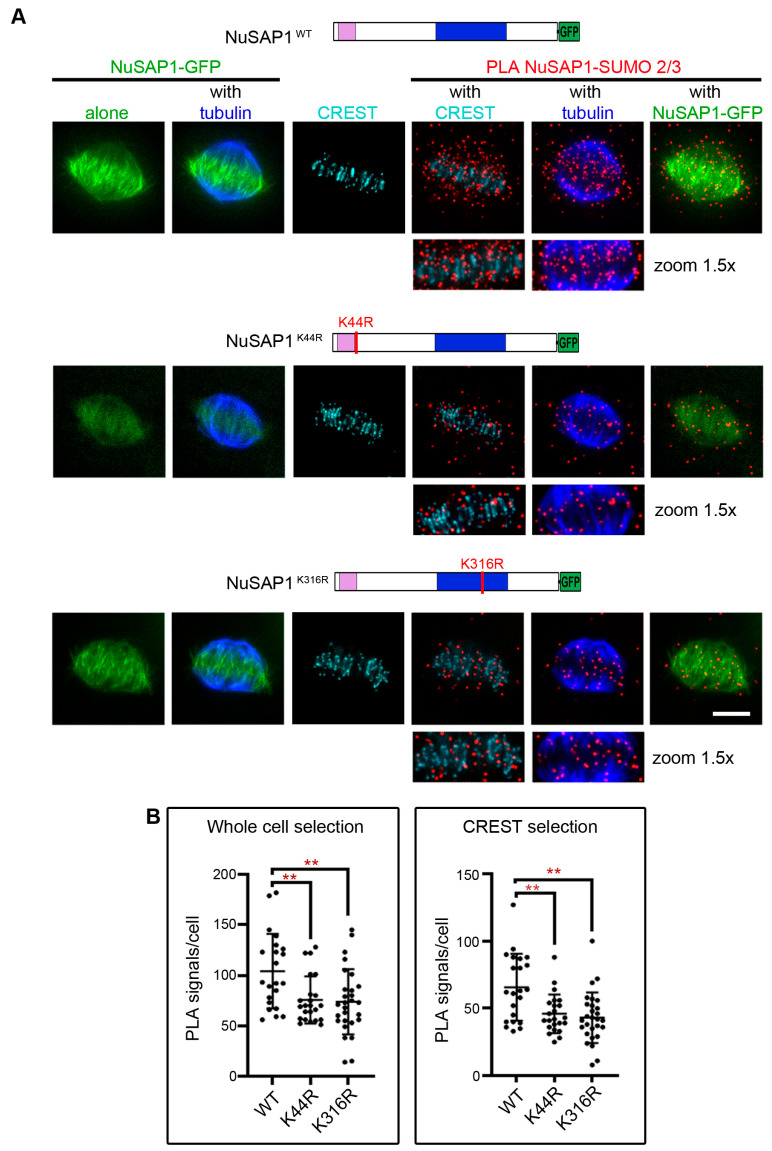
NuSAP1 mutation in putative SUMOylatable residues K44 and K316 impair the formation of PLA products with SUMO2/3. (**A**) Left: localization of the SAP and microtubule-binding domains in the NuSAP1. The red lines indicate putative SUMOylation sites identified in silico (K44 and K316). Right: the panels show NuSAP1-SUMO2/3 PLA signals in PHEM/Triton-solubilized cells expressing wild-type or mutant NuSAP1 versions (K44R and K316R) after endogenous NuSAP1 silencing. (**B**) Quantification of NuSAP1-SUMO2/3 PLA signals/cell: PLA signals significantly decrease in both mutant cell lines compared to WT, taking either the whole cell or the kinetochore/microtubule interface (CREST selection) as the RoI. A representative analysis is shown (at least 30 counted cells/sample, Mann–Whitney test, **, *p* < 0.01). The same trends were confirmed in an independent experiment with different basal intensity. Bar, 5 µm.

We generated NuSAP1 mutants lacking one or the other lysine and tested their PLA reactivity with SUMO2/3 peptides, as well their phenotypic effects. Codons encoding lysines K44 and K316 were mutagenized to arginine (R), so as to maintain the same charge as wild-type NuSAP1. We also introduced conservative mutations in all NuSAP1 sequences to render them resistant to NuSAP1-specific siRNAs, yet maintaining the same amino-acidic sequence as the endogenous protein, which enabled us to silence the endogenous gene and selectively analyze the effects of K44 or K316 mutant versions compared to wild-type, being siRNA-resistant. NuSAP1 versions were cloned in frame with an EGFP tag, downstream of a doxycyclin (dox)-inducible promoter as described [37]. After transfection in HeLa cells, cell lines with stably integrated wild-type NuSAP1 (NuSAP1^WT^) or mutant versions (lysine K44, NuSAP1^K44R^; or lysine K316, NuSAP1^K316R^) were selected (Figure S2B). All constructs were tested for PLA with SUMO2/3 in cells silenced for endogenous NuSAP1, using either straight PFA fixation or detergent treatment prior to fixation (Figure 4A). Automated quantification of PLA signals/cell revealed that both individual mutations at K44R and K316 significantly reduced NuSAP1-SUMO2/3 PLA product formation compared to NuSAP1^WT^ (Figure 4B), indicating that residues individually contribute to the formation of NuSAP1-SUMO2/3 products. Inspection of the PLA signal distribution showed that NuSAP1^WT^-SUMO2/3 accumulated at microtubule plus ends and at the interface with kinetochores (Figure 4A, top panel), reproducing the pattern of the endogenous protein. NuSAP1^K44R^ formed fewer PLA products with SUMO2/3, and those few PLA products localized predominantly along microtubules, whereas the kinetochore region appeared to be devoid of PLA signals (Figure 4A, middle panel). NuSAP1^K316R^-SUMO2/3 PLA products instead accumulated at kinetochores but were significantly less abundant at microtubules compared to NuSAP1^WT^ (Figure 4A, bottom panel). Thus, each one of the potentially SUMOylatable lysines identified in silico differentially contributes to NuSAP1 localization at kinetochores (K44) or at microtubules (K316). To corroborate these data, we examined the segregation of NuSAP1 mutants in fractionated protein extracts from mitotic synchronized cell lines (Figure S3): we found that NuSAP1^K44R^ decreased remarkably in the chromatin fraction, containing the histone-binding RCC1 protein, while NuSAP1^K316R^ showed a significant decrease in the tubulin-containing fraction. Thus, each lysine differentially affects NuSAP1 interactions either with chromatin (K44) or with microtubules (K316).

### 3.5. Separation of Function Analysis of NuSAP1 by Mitotic Phenotyping of Cell Lines Expressing K44 and K316 Mutants

**Figure 5:**
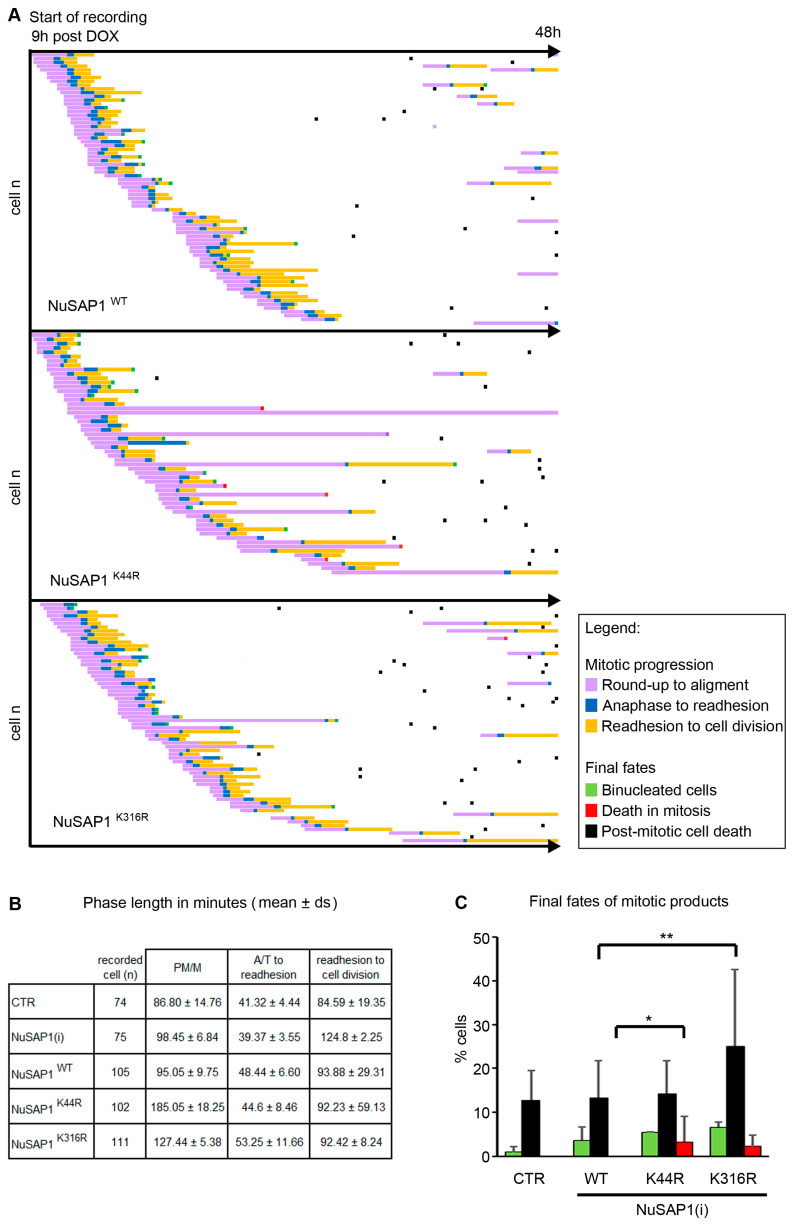
Videorecording of NuSAP1-GFP-expressing cell lines. (**A**) Single-cell analysis in cell lines expressing WT or mutant NuSAP1 versions in an endogenous NuSAP1-silenced background. Each line depicts one single cell arranged according to the timing of mitotic onset. Different colors depict mitotic stages, abnormalities and final fates (see legend). The length of each line indicates the duration of each phase. (**B**) Mean duration (min) of each phase in control cells (CTR) or in cells expressing NuSAP1^WT^ or mutant versions. (**C**) The histograms represent the frequency of the final fates of the cells after the first round of mitosis. At least 90 cells/sample were analyzed in 2 experiments. The *χ*^2^ test compares each class in mutant vs. NuSAP1^WT^. * *p* < 0.05; ** *p* < 0.01.

**Figure 6:**
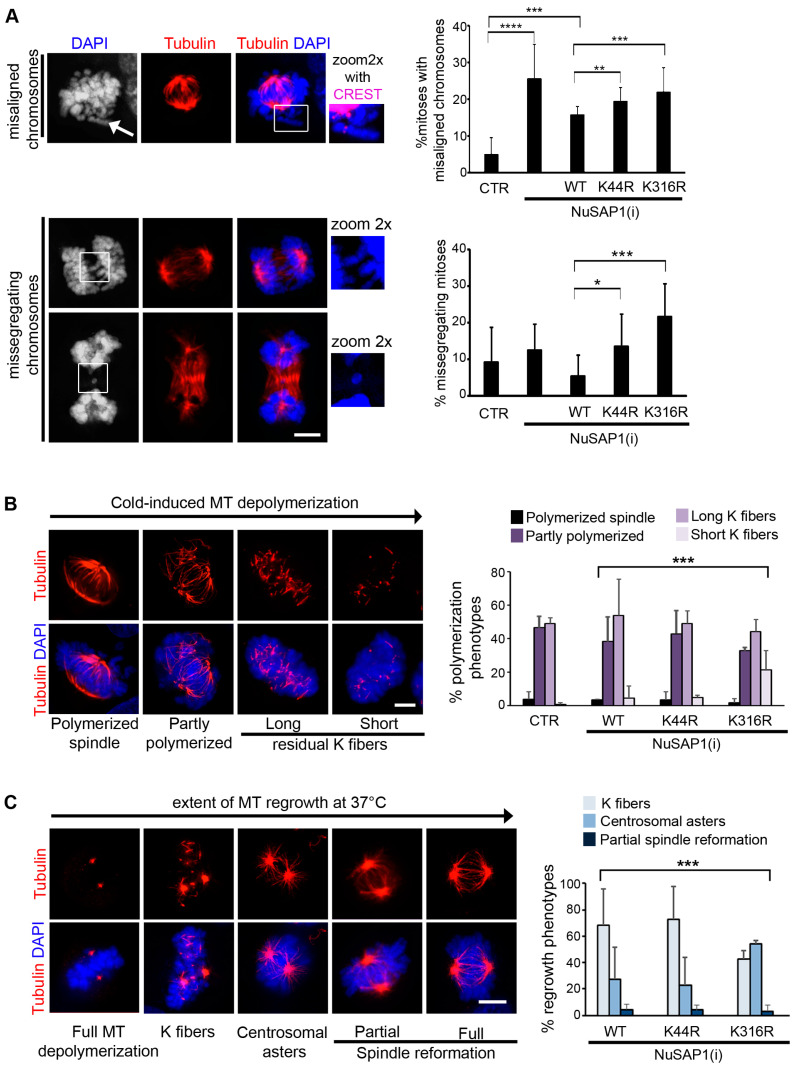
NuSAP1^K44R^ and NuSAP1^K316R^ induce distinct mitotic defects. (**A**) Mitotic abnormalities in cell lines expressing NuSAP1 mutants: upper panels, misaligned chromosome (arrowed). In the zoom-in (2x) of the boxed area, the chromosome is merged with CREST to stain kinetochores (magenta); bottom panels, mis-segregating chromosomes in anaphase and telophase. The histograms represent the frequency of abnormalities in cell lines expressing NuSAP1-GFP mutants. The *χ*^2^ test compares each class in mutant vs. NuSAP1^WT^-expressing cells (>300 analyzed cells, 4 experiments; * *p* < 0.05, ** *p* < 0.01, *** *p* < 0.001, **** *p* < 0.0001). (**B**) IF panels exemplify progressive stages of microtubule depolymerization observed after limited (15 min) incubation on ice. The graph quantifies the frequency of depolymerization phenotypes: NuSAP1^K316R^ yields a highly significant increase in advanced depolymerization (at least 170 analyzed mitoses/cell line, 2 experiments, multiple *χ*^2^ test; ***, *p* < 0.001). (**C**) Representative panels of progressive microtubule regrowth after extensive cold-induced depolymerization and incubation at 37 °C. Histograms represent regrowth phenotypes observed 1 min after T° up-shift in the indicated cell lines. A total of >100 analyzed cells/sample, 2 independent experiments, multiple *χ*^2^ test; *** *p* < 0.001. Bars, 5 µm.

The distinct localization of NuSAP1 mutants may yield different functional consequences. As a first approach to verify this, we decided to videorecord cell lines silenced for the endogenous NuSAP1 gene and reconstituted with NuSAP1^WT^, NuSAP1^K44R^ and NuSAP1^K316^ in time-lapse experiments to follow up mitotic and post-mitotic cell fates. Analysis of the videos considered every cell that reached round-up during the recording time (48 h from the moment of dox administration), which was followed up throughout the duration of the video. Examples of the videos obtained from these experiments are shown in Figure S4. Figure 5A shows the results of a representative experiment, in which single cells from each cell line are arranged according to their timing of mitotic entry (round-up) and changes are plotted over time (48 h). NuSAP1^WT^ cultures entered mitosis, progressed to align chromosomes and divided with a similar timing to that of cultures treated with neutral siRNA (Figure 5B). Some cells re-entered a second mitotic round and progressed normally during the remainder of the video. NuSAP1^K44R^-expressing cultures showed instead a significantly prolonged duration from round-up to alignment (Figure 5A). In some cells, the length of that window occupied most of the video, such that very few cells re-entered a second mitotic round. In some cases, cell death was induced during prolonged prometaphase (an example of mitotic cell death during mitotic delay is shown in Figure S4, and the frequency of these events is represented by red squares in Figure 5A). In parallel, we fixed and stained cell samples in order to analyze mitotic figures in higher resolution. We detected chromosome misalignment in about 20% of NuSAP1^K44R^ metaphases; past that stage, chromosome mis-segregation fell below 7% of late mitotic cells (Figure 6A). These data suggest that NuSAP1^K44R^-induced defects were sensed by the SAC, resulting either in defect correction or in cell death induction during mitotic delay.

NuSAP1^K316R^ cell lines displayed a distinct behavior: most cells progressed through mitosis and divided with no significant delay, except in rare cases. Some cells re-entered a second round of mitosis during the recording time. In most cells, however, cell death was significantly induced in the interphase that followed mitotic exit (exemplified in Figure S4; this cell fate is represented by black squares in Figure 5A and quantified in Figure 5C). In IF assays, NuSAP1^K316R^-expressing cells displayed chromosome misalignment in metaphase and a highly significant frequency of chromosome mis-segregation in anaphase and telophase (Figure 6A). These data suggest that cells expressing NuSAP1^K316R^—which showed a defective localization at microtubules (Figure 4) and a low co-fractionation with tubulin (Figure S3)—induced functional defects that were not sensed by the SAC, did not delay anaphase onset and originated significant chromosome mis-segregation in daughter cells (Figure 6A), often followed by post-mitotic death (Figure 5).

### 3.6. Mutation of K316 Accelerates Mitotic Spindle Disassembly and Impairs Microtubule Regrowth

At this point, we asked whether NuSAP1 mutation in either K44 or K316 affected microtubule function. We first compared cold-induced microtubule depolymerization in cell lines expressing either wild-type or mutant NuSAP1 after endogenous NuSAP1 silencing. After limited incubation at 0 °C (15 min), cells were fixed and stained. Exemplifying panels of the state of microtubules under these conditions are shown in Figure 6B. In control cultures expressing endogenous NuSAP1, essentially two types of mitotic figures were depicted with practically equivalent frequency: cells displaying a still recognizable spindle structure with few, thin microtubules (defined as partly polymerized), and cells in which the spindle structure was substantially lost, the poles were unstructured and only long tubulin fibers remained (categorized as long fibers). Cell lines expressing NuSAP1^WT^ or NuSAP1^K44R^ showed very similar patterns to that of cells expressing native NuSAP1. In the NuSAP1^K316R^-expressing cell line, the frequency of these phenotypes decreased and a third phenotype appeared, represented by cells displaying very short tubulin fragments, indicating a more advanced stage of depolymerization. Thus, expression of NuSAP1^K316R^, which associates weakly with tubulin, accelerates microtubule depolymerization.

We next analyzed the effect of NuSAP1 mutants on microtubule regrowth after extensive depolymerization (40 min at 0 °C) and rewarming to 37 °C. Figure 6C shows representative images of progressive microtubule regrowth up to complete spindle reformation. In this assay, microtubules are initially nucleated from kinetochores, while centrosomal nucleation is resumed with somewhat slower kinetics; eventually both types of microtubules merge in a functional spindle [48,49]. In all cell lines, only a negligible fraction of the cells showed a partially reformed spindle 1 min after up-shift to 37 °C. In both NuSAP1^WT^ and NuSAP1^K44R^ cell lines, most mitotic cells displayed kinetochore-nucleated microtubules, while about 20% of mitoses had also resumed centrosomal nucleation by this time. In contrast, microtubule regrowth from kinetochores (K fibers) was inhibited in NuSAP1^K316R^ cells, while microtubule regrowth from centrosomes was not significantly affected: the proportion between kinetochore and centrosomal microtubules was therefore reversed in NuSAP1^K316R^ compared to both wild-type and NuSAP1^K44R^ cell lines, indicating a differential importance of NuSAP1^K316^ in the kinetochore-driven vs. the centrosome-driven spindle assembly pathways.

## 4. Discussion

Spatial control of mitotic regulatory factors represents a fundamental level of control of the mitotic spindle organization, function and hence of chromosome segregation. In this work, we have sought to gain spatial information on the microtubule regulatory factor NuSAP1 during mitosis. We report two novel findings: first, RANBP2 contributes to regulate NuSAP1 abundance and localization at mitotic microtubules plus ends, where NuSAP1 can exert its microtubule-stabilizing functions [2,3,4,5] during chromosome alignment and segregation. RANBP2 is also required to stabilize NuSAP1-SUMO2/3 PLA products at microtubule plus ends and at the interface with kinetochores. Second, we find that these products involve lysine residues in two newly identified SUMO consensus sites, the mutation of which differentially affect distinct NuSAP1 functions.

Analysis of the temporal distribution of NuSAP1-RANBP2 PLA signals (Figure 1) showed that they specifically formed in mitosis but not in interphase and peaked in late prometaphase and metaphase. The finding that RANBP2 silencing decreased NuSAP1 abundance in Western assays of mitotic cell extracts, counteracted by inhibiting the proteasome activity (Figure 2A–C), implicates RANBP2 in modulating NuSAP1 turn-over during mitosis. In support of that view, IF assays, after pre-extracting the cells using a protocol that stabilizes microtubule-associated proteins, showed a requirement for RANBP2 in stabilizing NuSAP1 at microtubule plus ends in phases in which microtubules are engaged in chromosome capture. At that time, CDK1 is highly active and is reported to phosphorylate NuSAP1, which lowers its affinity for microtubules [43], preventing their hyperstabilization during the search-and-capture process. Aurora A [50] and ATM [51] kinases also phosphorylate NuSAP1 and might contribute to modulate NuSAP1′s affinity for tubulin. After microtubule/kinetochore attachments are established and the SAC is released, NuSAP1 is dephosphorylated, which is reported to increase its binding to microtubules [43]. We find that the protective effect of RANBP2 becomes dispensable at this point (Figure 2D).

The role of RANBP2 identified in this work adds specificity and fine-tuning to control of NuSAP1 stability during mitosis. As summarized in the introduction, NuSAP1 has critical microtubule regulatory functions. In early mitosis, an SCF-type ligase ensures moderate NuSAP1 turn-over during spindle assembly [52]. Later, APC/C-dependent ubiquitination in telophase and cytokinesis conveys NuSAP1 to massive degradation [11]. Microtubules themselves shield NuSAP1 from ubiquitination [12]. Importin vectors contribute to anchor NuSAP1 to microtubules during mitosis, thus preventing its premature ubiquitination-dependent degradation [11]. At the same time, however, their binding inhibits NuSAP1 activity in microtubule elongation and function [2,3]. Thus, NuSAP1 function is tuned by a subtle balance between being importin-bound and protected, but functionally inactive, and being released from import vectors and functionally proficient, yet becoming exposed to ubiquitination and degradation. Importin beta localizes to the spindle microtubules with the highest concentration at poles and a progressive dilution towards microtubule plus ends [53], the opposite to that of NuSAP1. RANBP2 interacts with microtubules throughout their length and a fraction is recruited to microtubule-attached kinetochores by the RANGTP-dependent export vector CRM1 [37,54]. RANBP2 can bridge NuSAP1 to microtubule plus ends, where the concentration of importin beta is low and the concentration of RANGTP is high [55,56,57], adding a further layer of control of NuSAP1 stability in prometaphase and metaphase.

RANBP2 also stabilizes NuSAP1-SUMO2/3 PLA products (Figure 3). Direct attempts to recover NuSAP1 from SUMO pulldown experiments were unsuccessful, both in previous studies [13] and in our laboratory. Under the same conditions, we recovered SUMO-RANGAP1, which has an abundant SUMOylated fraction throughout the cell cycle. NuSAP1-SUMO2/3 products, instead, form in a limited cell cycle window and at restricted locations in mitotic cells. Given that these limitations evidently hinder the biochemical detection of SUMOylated NuSAP1, we searched for candidate SUMO sites in the NuSAP1 (Figure S2A) and focused on two lysines in the SAP DNA-binding domain (K44) and the microtubule-binding domain (K316), respectively. We found that mutation of each lysine differentially affected NuSAP1 localization, yielding distinct functional effects.

Mutation of K44 impaired NuSAP1-SUMO2/3 localization at kinetochores (Figure 4A) and weakened NuSAP1 association with chromatin (Figure S3). These data, coupled with videorecording assays, suggest that the decreased association of NuSAP1^K44R^ with kinetochores underlies the severe delay in achieving chromosome alignment observed in NuSAP1^K44R^ cells (Figure 5 and Figure S4). In a fraction of cells, mitotic death was triggered during that delay. Cells that progressed further harbored a relatively low frequency of chromosome mis-segregation at mitotic completion (Figure 6A), indicating that K44 hinders chromosome alignment, yet does not impair the misattachment correction system during mitotic delay.

K316 falls in the microtubule-binding domain of NuSAP1. A K316 mutation is reported in two databases (UniProt and the genome aggregation database gnomAD [58]) as a K-to-I substitution, as a rare variant of unclassified clinical significance. Characterizing this mutant was therefore of particular interest. We found that mutation of K316 decreased both NuSAP1-SUMO2/3 localization at microtubules and NuSAP1 association with the tubulin-containing fraction (Figure 4 and Figure S3). The reduction in NuSAP1′s stabilizing activity at microtubules can account for two distinctive defects in NuSAP1^K316^ cells: accelerated microtubule depolymerization (Figure 6B) and impairment of the kinetochore-driven pathway of spindle regrowth (Figure 6C), in which NuSAP1 exerts a relevant function particularly during early embryogenesis [59]. During spindle assembly, NuSAP1, as well as HURP, are required for the kinetochore-driven microtubule nucleation pathway under RANGTP control [57,60], while the centrosome-driven pathway is more dependent on pericentrin, PLK-1, TPX2 and Aurora-A. Our data indicate that K316 is important for NuSAP1 function in the kinetochore pathway. A subset of colon cancers was reported to overexpress RANBP2, which modulated sensitivity to certain anti-microtubule drugs [61]. Interestingly, in that study, RANBP2 silencing impaired microtubule regrowth from kinetochores, but not from centrosomes, similar to the effect characterized here for NuSAP1^K316R^. NuSAP1^K316R^ did not significantly prolong prometaphase (Figure 5), suggesting that its effects were not sensed by the spindle checkpoint system, possibly because this mutation does not impair NuSAP1 localization at kinetochores, where the SAC components reside. Mitotic defects remained uncorrected, yielding significant segregation errors (Figure 6) and death of the resulting daughter cells in the next interphase (Figure S4).

**Figure 7:**
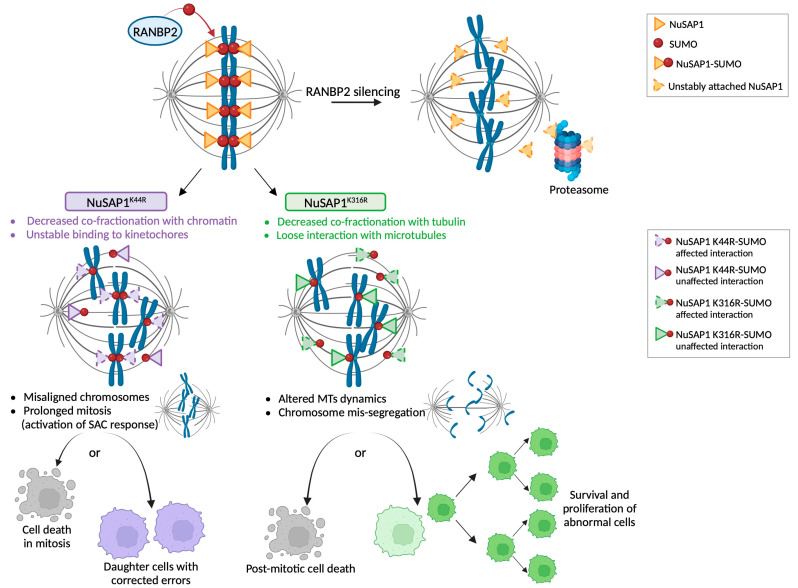
NuSAP1 mutants reveal distinct mitotic functions of NuSAP1. The model proposes that RANBP2 stabilizes NuSAP1 levels and NuSAP1-SUMO2/3 interactions with microtubules and kinetochores. Below, the mutations of K44 in the SAP domain and of K316 in the microtubule-binding domain destabilize specific sets of NuSAP1 interactions, i.e., with kinetochores, and with microtubules, respectively, and hence generate distinct mitotic defects that evolve into different cell fates of mitotic products. The model was created using BioRender.com.

These results provide a separation-of-function analysis of NuSAP1 and pinpoint the specificity with which NuSAP1 mutation in one or the other SUMO consensus sites identified in silico affects mitosis, eliciting differential phenotypes and distinct fates in the cellular progeny. A hypothetical model based on these findings is proposed in Figure 7. These data add novel information on NuSAP1 regulation and function and, more generally, hint at a role of SUMOylation in modulating the spatial and temporal activity of factors implicated in mitotic spindle control.

## Data Availability

All data are available from the authors upon request.

## Supplementary Materials

The following supporting information can be downloaded at: https://www.mdpi.com/article/10.3390/cells12212545/s1. Table S1, Primary antibodies used in this work. Figure S1. Different fixation protocols reveal distinct NuSAP1 fractions. Figure S2. Expression of EGFP-tagged NuSAP1^WT^, NuSAP1^K44R^ and NuSAP1^K316^ proteins from doxycycline (DOX)-inducible epB-Bsd-TT EGFP vector in HeLa cells. Figure S3. Segregation of NuSAP1^WT^, NuSAP1^K44R^ and NuSAP1^K316R^ in fractionated mitotic protein extracts. Figure S4. Videorecording protocol and exemplifying frames from typical videos.
