# Supplementary material for: Distinct Mitotic Functions of Nucleolar and Spindle-Associated Protein 1 (NuSAP1) Are Controlled by Two Consensus SUMOylation Sites
Source: Cells. 2023 Oct 30;12(21):2545. doi: 10.3390/cells12212545 (PMC10650578; doi:10.3390/cells12212545)
Supplement: Supplementary file 1 [file cells-12-02545-s001.zip › cells-2673198-supplementary.pdf]

Distinct Mitotic Functions of Nucleolar and Spindle-Associated Protein 1 (NuSAP1) Are Controlled by Two Consensus SUMOylation Sites

Michela Damizia [1,2,3], Ludovica Altieri [1,2], Vincenzo Costanzo [1] and Patrizia Lavia [1,*]

[1] Institute of Molecular Biology and Pathology (IBPM), CNR National Research Council of Italy, 00185 Rome, Italy; michela.damizia@unitn.it (M.D.); ludovica.altieri@uniroma1.it (L.A.); vincenzo.costanzo@ibpm.cnr.it (V.C.)
[2] Department of Biology and Biotechnology "Charles Darwin", Sapienza University of Rome, 00185 Rome, Italy
[3] Department of Cellular, Computational and Integrated Biology (CIBIO), University of Trento, 38123 Trento, Italy
* Correspondence: patrizia.lavia@cnr.it or patrizia.lavia@uniroma1.it

Table S1. Primary antibodies used in this work

Protein	Host	Source	Catalog	IF or PLA dilution	Western dilution
Actin	Goat	Santa Cruz	sc-1862	-	1:200
Alpha tubulin	Mouse	Sigma Aldrich	T5168	1:1500	1:1000
Alpha tubulin	Chicken	Abcam	ab89984	1:100	-
CREST	Human	AntibodiesInc	15-234-0001	1:30	-
GAPDH	Mouse	Santa Cruz	sc-32233		1:2000
EGFP	Mouse	Selleck	A1073	-	1:1000
NUP153	Mouse	Abcam	Ab24700	-	1:500
NuSAP1	Rabbit	Abcam	ab93779	1:200	1:2000
NuSAP1	Rabbit	Proteintech	12024-I-AP	1:150	1:1500
RANBP1	Goat	Santa Cruz	sc-28576	-	1:250
RANBP2	Mouse	Santa Cruz	sc-74518	1:50	1:50
RANBP2	Rabbit	Abcam	ab64276		1:100
RANGAP1	Mouse	Santa Cruz	sc-28322	1:100	-
RCC1	Goat	Santa Cruz	sc-1161	-	1:200
SUMO2/3	Mouse	MBLI	M114-3	1:200	_
TOP2A	Rabbit	TopoGEN	TG2011-1	-	1:1000

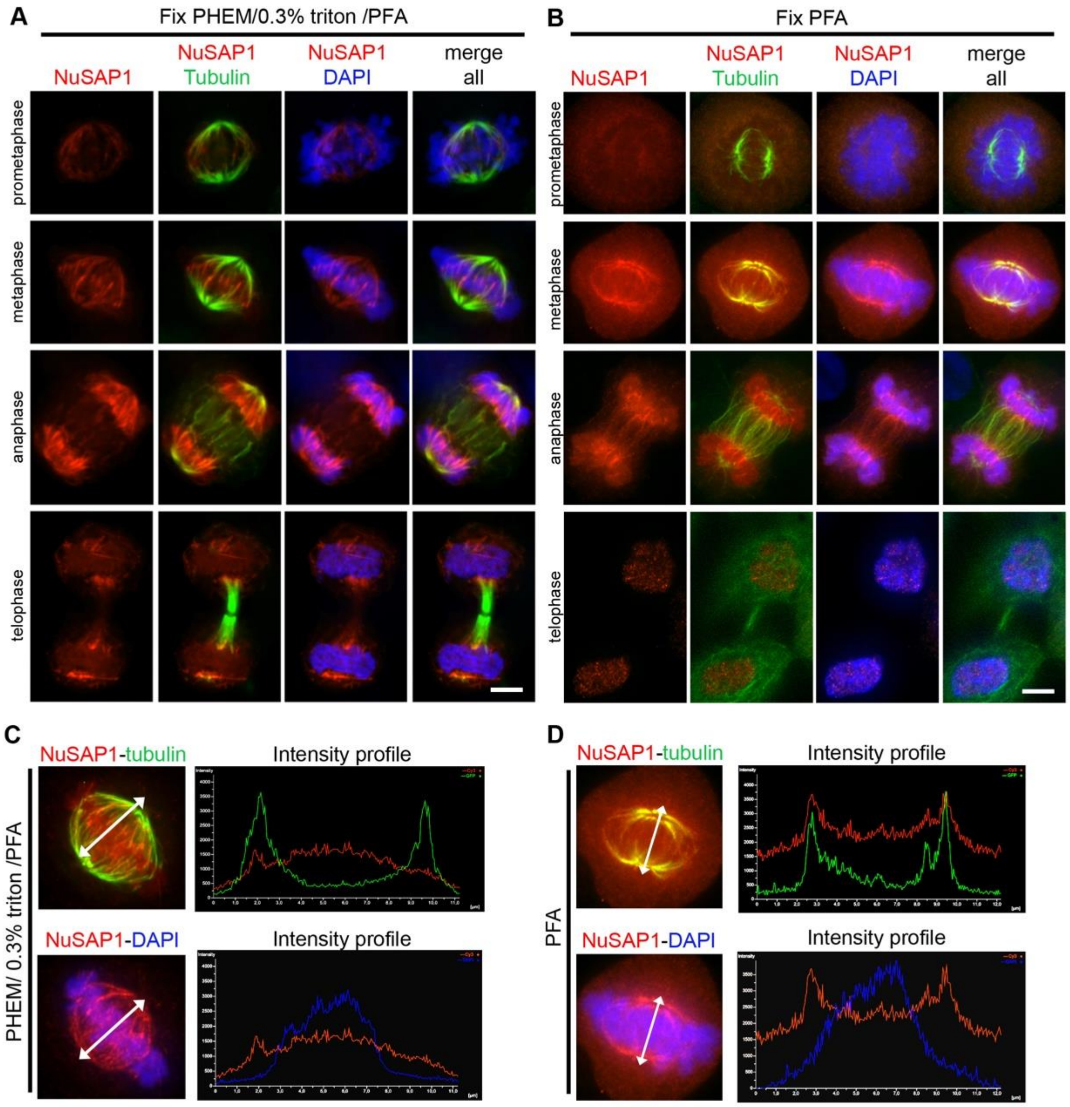

Figure S1. Different fixation protocols reveal distinct NuSAP1 fractions. Exemplifying IF panels show NuSAP1 (red), tubulin (green) and DNA (DAPI, blue. **(A**) Cells were treated with PHEM supplemented with 0.3% Triton X-100 to extract soluble proteins prior to fixation in 3.7% paraformaldehyde (PFA). **(B)** Cells were fixed directly in PFA. **(C-D)** The graphs show the intensity profile of the NuSAP1 signal plotted vs. either tubulin (green) or DAPI (blue) intensity profiles in exemplifying metaphases. In **(C),** detergent-resistant NuSAP1 (PHEM/0.3%Triton, then PFA) has highest intensity in the spindle central region defined by the poles (highest tubulin intensity), overlapping the aligned chromosome region (highest DAPI signal intensity), indicating that under this condition NuSAP1 is predominantly concentrated at microtubule plus-ends. In **(D),** direct PFA fixation visualizes the entire cellular protein pool. Under this condition NuSAP1 was also visible along the spindle microtubule length, including around the spindle poles. With both procedures, a decline in NuSAP1 abundance can be appreciated in late telophase due to regulated ubiquitin/proteasome degradation (details in main text). Bars, 5 μm.

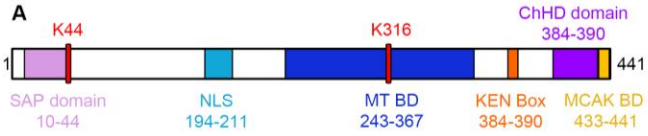

Putative SUMOylatyon sites

Position	Peptide	Type	P-value
44	KALKGYIKHEARKGN	SUMOylation consensus	0.005
316	TVSGGTPKGEAVLGT	SUMOylation consensus	0.037

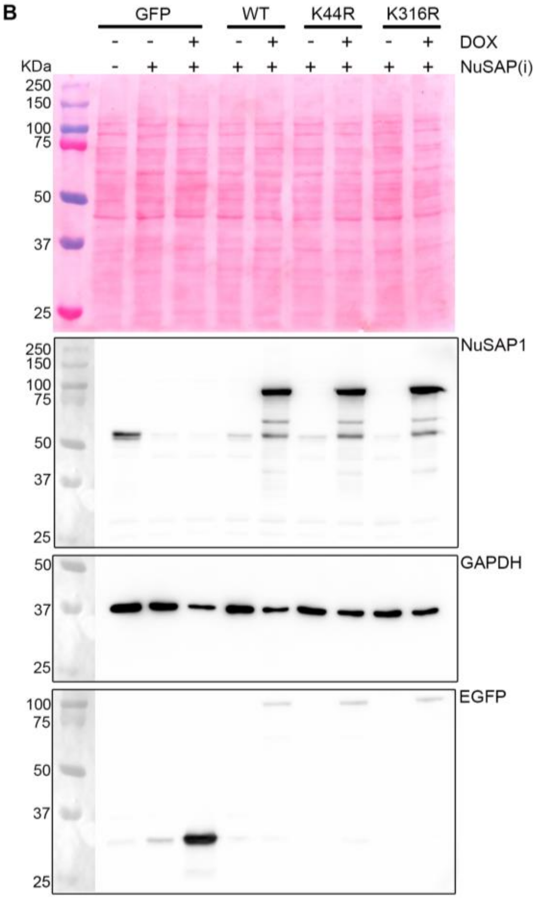

Figure S2. Expression of EGFP-tagged NuSAP1^{WT}, NuSAP1^{K44R}, and NuSAP1^{K316} proteins from doxycycline (DOX)-inducible epB-Bsd-TT EGFP vector stably integrated in HeLa cells (details in Materials and Methods, main text). **(A)** Schematic map of the NuSAP1 protein: the positions (amino acid numbering) indicate relevant functional regions or domains (NLS, nuclear localization signal; BD: binding domain; ChHD charged helical domain). Red bars mark the position of putative lysine SUMO acceptor residues identified *in silico* using two independent SUMO prediction bioinformatic tools (SUMOplot and GPS-SUMO). P-value scores according to GPS-SUMO are indicated. **(B)** Western blot assay of cell extracts from uninduced (-) and dox-induced (+) cell lines with integrated NuSAP1-EGFP constructs in WT and mutant versions. After blasticidin selection, stable integrant cell lines were treated with NuSAP1-specific siRNAs, indicated as NuSAP(i). A cell line with integrated empty vector (indicated as GFP) was used for control. After transfer, the filter was incubated with anti-NuSAP1 and anti-EGFP to verify DOX induction and RNAi-resistance of the constructs and anti-GAPDH for protein loading (digital acquisition, Azur Biosystems).

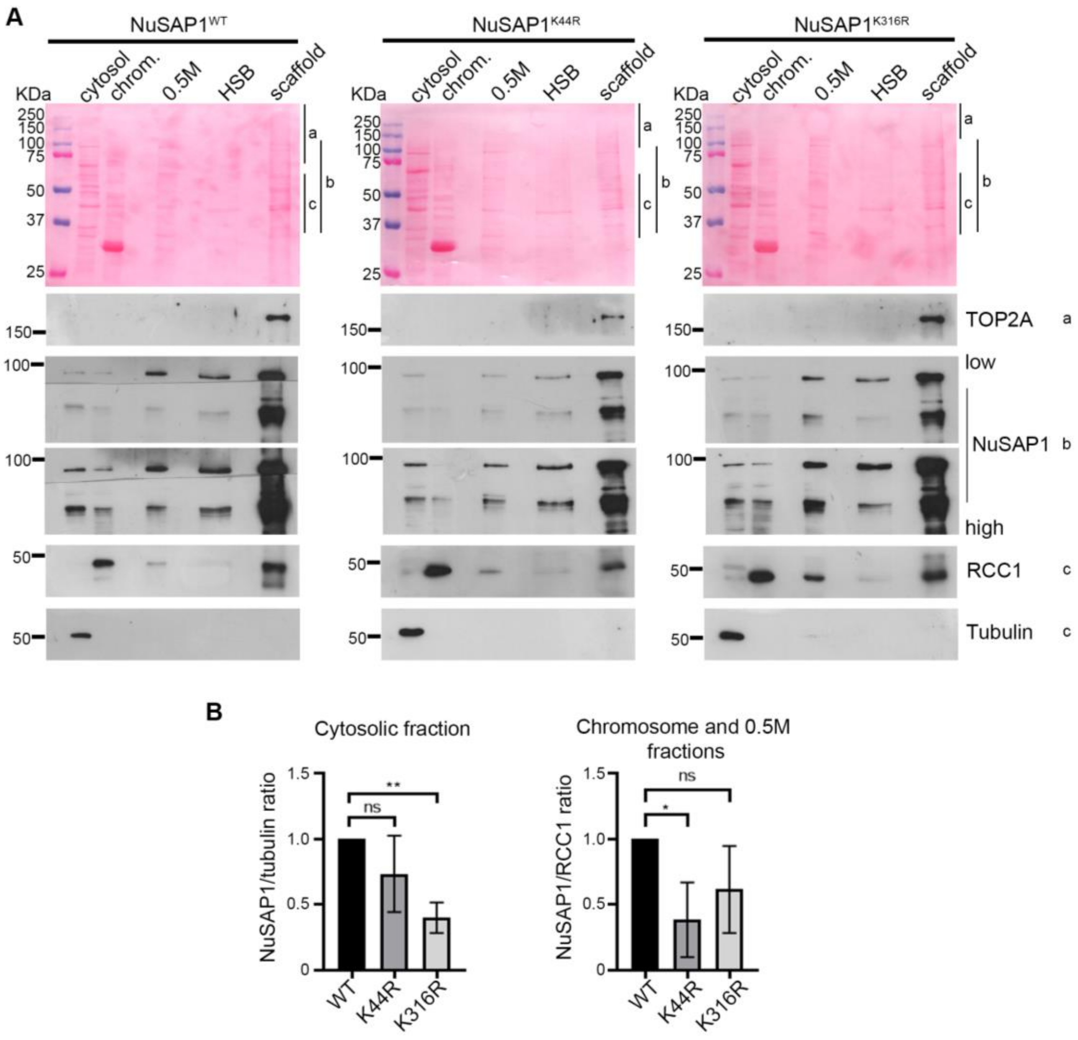

Figure S3. Segregation of NuSAP1^{WT}, NuSAP1^{K44R} and NuSAP1^{K316R} in fractionated mitotic protein extracts. (**A**) SDS-PAGE and filters from fractionated mitotic extracts from NuSAP1^{WT}-, NuSAP1^{K44R}- and NuSAP1^{K316R}-expressing cell lines after endogenous NuSAP1 silencing and synchronization in mitosis (Materials and Methods in main text). After electrophoresis through 11% SDS-PAGE and protein transfer to NTC membranes, the filters were cut (square brackets) and the resulting filter portions were incubated wih antibodies as follows in order to probe the extract fractions: first cut above 100 kDa (*a*), to incubate with anti-topoisomerase 2 alpha antibody (TOP2A) for the chromosome scaffold fractions (in this example the wild-type filter was inadvertently cut at the edge of the closely migrating 75 kDa band); filter portion (*b*) was incubated with rabbit NuSAP1 antibody (Proteintech 12024-I-AP); a low exposure (for the scaffold fraction) and a higher exposure are shown. The filter was cut again below the 75 kDa marker (*c*) and sequentially incubated with goat anti-RCC1 (histone-interacting) for chromatin and 0.5M HSB fractions, and with mouse anti-tubulin (cytosolic fraction). (**B**) The histograms represent the ratio of NuSAP1-GFP co-fractionating with either tubulin (left), or RCC1 (right), in NuSAP1^{WT}, NuSAP1^{K44R} and NuSAP1K^{316R} cell lines (3 repeats). The ratio of NuSAP1^{WT}/marker (RCC1, or tubulin) is taken as 1. The NuSAP1^{K44R} signal significantly decreases in the chromatin fractions, while NuSAP1^{K316R} decreases highly significantly in the tubulin-containing fraction. Student-t test; ns, not significant; *$p<0.05$; **$p<0.01$.

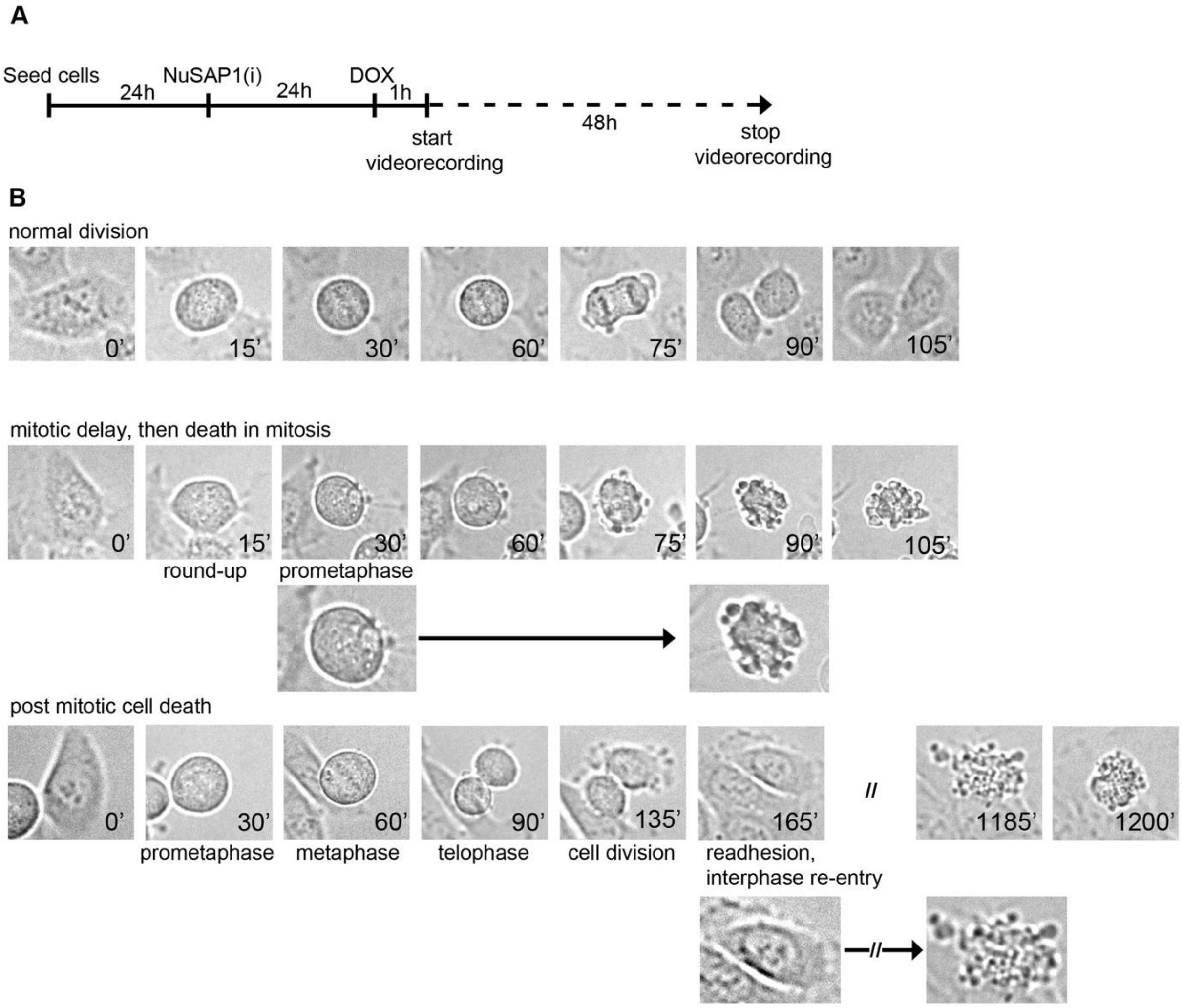

Figure S4. Videorecording protocol and exemplifying frames from typical videos. (A). The protocol shows the schedule for siRNA-mediated knockdown of endogenous NuSAP1 and doxycycline (DOX)-dependent induction of NuSAP1-GFP in cell lines expressing wild-type or mutant forms. Videorecording starts1 h after DOX administration. (B). Still frames from videorecorded cells showing representative examples of cell fates. For ease of classification, the frame immediately preceding round-up was conventionally timed as time 0. DIC frames are taken every 15 min: thus, videos were constructed from 193 frames for each cell (Nikon software NIS-Elements 3.1). Only the most significant frames are shown. Upper panel: a cell undergoing normal division through classical stages of mitotic progression; in this example the cell entered prometaphase (time 15′) and reached full chromosome alignment (metaphase) at time 60′; anaphase was already ongoing in the next time frame (75′). Cell division was complete by 90′ and the two newly generated daughter cells readhered by 105′ (most frequently, but not exclusively, seen in the NuSAP1^{WT} cell line). Middle panel: a cell entered prometaphase (30′), but seemed to arrest in that stage and failed to achieved chromosome alignment; by 75′ signs of chromatin condensation and cell shrinkage appeared; by 90′ the mitotic death pathway was evidently activated (most frequently, but not exclusively, seen in the NuSAP1^{K44R} cell line). Lower panel: a cell going through prometaphase (30′), apparently normal metaphase (60′), telophase (90′) and cell division (135′). The daughter cells seemed to re-establish normal adhesion. However, around 18 h later, the adherent cell underwent an evident process of cell death; in the 1185′ frame, severe signs of aberrant chromatin condensation and fragmentation were evident (most frequently, but not exclusively, seen in the NuSAP1^{K316R} cell line). The frequency of mitotic phenotypes and final cell fates are shown in Figure 5.
